# Cross-Sectional Study on the Sero- and Viral Dynamics of Porcine Circovirus Type 2 in the Field

**DOI:** 10.3390/vaccines8020339

**Published:** 2020-06-26

**Authors:** Chao-Nan Lin, Ni-Jyun Ke, Ming-Tang Chiou

**Affiliations:** 1 Animal Disease Diagnostic Center, College of Veterinary Medicine, National Pingtung University of Science and Technology, Pingtung 91201, Taiwan; conny1668@gmail.com; 2 Department of Veterinary Medicine, College of Veterinary Medicine, National Pingtung University of Science and Technology, Pingtung 91201, Taiwan

**Keywords:** porcine circovirus type 2, porcine circovirus-associated diseases, ELISA, real-time PCR

## Abstract

Porcine circovirus-associated diseases (PCVADs) cause considerable economic losses in industrial pork production in the field. To minimize the economic losses due to PCVAD, porcine circovirus type 2 (PCV2) vaccines have been developed, and there is widespread vaccination worldwide today. However, limited information is available concerning the current status of PCV2 infection in the field on the Asian continent. The present study aimed to assess sero- and viral dynamics of PCV2 from 12 PCV2-contaminated pig herds with vaccination against PCV2 in Southern and Central Taiwan. In particular, the level of PCV2 load during the window period for seroconversion using real-time polymerase chain reaction and a commercial enzyme-linked immunosorbent assay (ELISA) kit. Our results revealed that pig herds showed slight or no seroconversion after three to four weeks post-PCV2 immunization. The presence of PCV2 was observed during the window period for seroconversion in all herds. In conclusion, natural exposure of PCV2 occurs in the growing to fattening period, and viremia can last until slaughter. Additionally, our findings indicate that using ELISA showed the level of antibodies and aided in the understanding and surveillance of the current PCV2 status in the field.

## 1. Introduction

Porcine circovirus type 2 (PCV2) is one of the major primary infectious agents as contributing to mortality in porcine respiratory disease complex (PRDC) [1]. PCV2 is a nonenveloped, circular, single-stranded DNA virus belonging to the genus Circovirus, family *Circoviridae* together with the genus Cyclovirus [2]. PCV2 is the causative agent of porcine circovirus-associated diseases (PCVADs), which have caused enormous economic losses in industrial pork production [1]. Most of the time the infection is subclinical but in some circumstances such as coinfections with other respiratory pathogens it can cause the postweaning multisystemic wasting syndrome, clinically characterized by wasting respiratory disease and enteritis [3].

To minimize the economic losses of PCVAD, vaccines have been developed and are widely used so far. All commercially available PCV2 vaccines, including subunit, inactivated whole virus, or inactivated chimeric virus, can induce not only humoral immune response but also cellular immunity [4,5,6,7]. Under experimental or farm conditions, a significant increase in the average daily weight gain and a decrease in the mortality rate were observed in all PCV2 vaccine trials [8]. Thus, continued PCV2 vaccination may lead to PCV2 elimination [9,10]. However, several disadvantages of PCV2 vaccines are concerning, including interference of maternal-derived antibody (MDA) [4,6,7,11,12,13,14] and age-related factors [12] that can potentially interfere with the efficacy of the subunit and inactivated whole virus PCV2 vaccines. A previous study showed that a significant negative correlation between the level of PCV2 MDA and the level of PCV2 antibodies at 21 days post-vaccination with inactivated whole virus vaccine [11]. Additionally, pigs with a low sample to positive (S/P) ratio at the moment of vaccination had a better growth performance than those with high PCV2 MDA values [15].

PCV2 is an important pathogen in most pig-producing countries, with an observed high prevalence of this disease in Taiwan [16,17]. A commercial subunit PCV2 vaccine (Ingelvac CircoFLEX^®^) was first introduced to Taiwan in September 2010. The PCV2 vaccine has been highly effective in reducing clinical signs of PCVADs and improving production since vaccination was widely implemented in Taiwan. However, the number of PCVAD cases appeared to increase in growing pigs based on data from the Animal Disease Diagnosis Center (ADDC) of National Pingtung University of Science and Technology (NPUST) in recent years [18]. Additionally, gilts and sows in pig herds have been exposed to field virus in the fattening stage or had PCV2 vaccines when they were 3 weeks old since widespread vaccination began almost a decade ago. Therefore, the sows and their offspring have varied levels of PCV2 antibodies [14]. To date, all international commercially available vaccines have been developed in the United States or European counties [19], and limited information is available concerning the sero- and viral dynamics of the current status of PCV2 infection in the field on the Asian continent. Herein, this retrospective study aimed to elucidate the sero- and viral dynamics of PCV2 after PCV2 immunization in the field in Taiwan. The study was performed in 12 PCV2-contaminated pig herds in Southern and Central Taiwan.

## 2. Materials and Methods 

### 2.1. Sample Source and Processing

A cross-sectional study was conducted in Southern and Central Taiwan, and a total of 875 blood samples (ADDC, College of Veterinary Medicine, NPUST, Pingtung, Taiwan), from 12 PCV2-contaminated pig herds were collected by jugular venipuncture from pigs and submitted to the ADDC of NPUST for diagnostic or routine monitoring purposes. The serum samples were carefully transferred into 1.5 mL centrifuge tubes after centrifugation at 2150× *g* for 15 min. The stock serum was kept at −80 °C until needed. Details of the case numbers in the present retrospective study are shown in Table S1 (Supplementary Material). The overview of pig herd characteristics in this retrospective study is summarized in Table 1.

### 2.2. Total Nucleic Acid Extraction and PCV2 Real-Time Polymerase Chain Reaction (PCR)

Total nucleic acids were extracted from serum samples using a MagNA Pure LC total nucleic acid isolation kit (Roche Diagnostics GmbH, Mannheim, Germany) operated on a MagNA Pure LC 2.0 automatic nucleic acid extraction instrument (Roche Diagnostics GmbH, Mannheim, Germany), according to the manufacturer’s instructions. To quantify the PCV2 load in serum samples, quantitative real-time PCR assay was tested and performed with a LightCycler^®^ 96 system (Roche Applied Science, Basel, Switzerland) as described Tsai et al. [18].

### 2.3. Serology

All serum samples were tested for the presence of anti-PCV2 antibodies using a commercial enzyme-linked immunosorbent assay (ELISA) kit (Porcine Circovirus type 2 Antibody Test, BioChek B.V., Reeuwijk, Holland) according to the manufacturer’s instructions. Serum samples were considered positive for the presence of PCV2 antibody when the average S/P ratio was ≥0.5.

### 2.4. Statistical Analysis

Student’s *t*-test using Prism 8.0 (GraphPad Software, La Jolla, CA, USA) was used to assess differences in the presence of PCV2 MDA from suckling piglets born from different sow vaccination programs against PCV2. *p* values <0.05, <0.01, and <0.001 were considered statistically significant, highly significant, and very highly significant, respectively.

## 3. Results

### 3.1. Passively Acquired PCV2 Antibodies of Piglets from Different Sow Vaccination Programs

**Figure 1:**
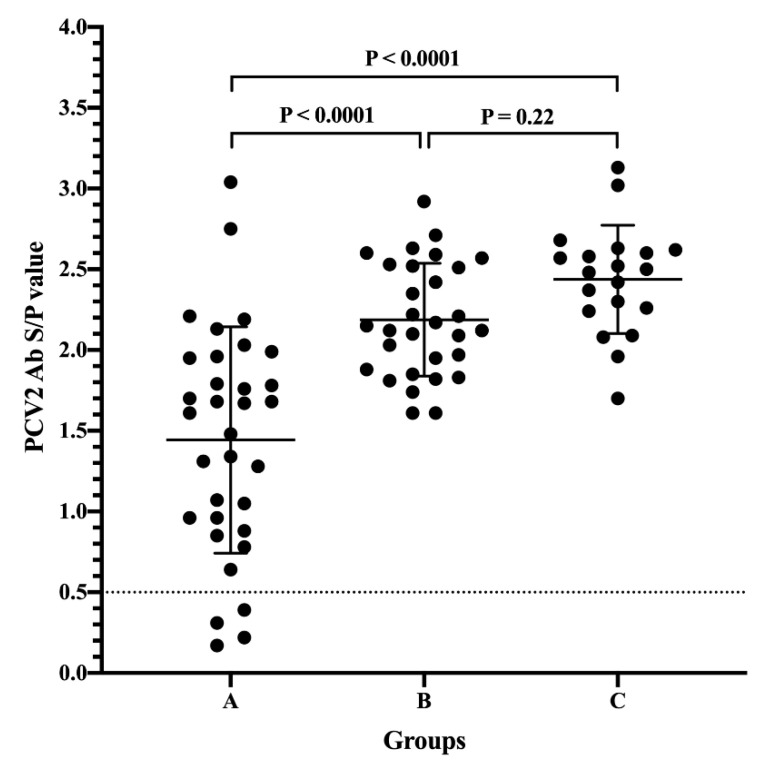
Comparison of maternally derived antibodies (MDA) of porcine circovirus type 2 (PCV2) on suckling piglets (only from 3 to 4-week-old piglets without PCV2 vaccination) born from different sow immunization programs against PCV2. A: sows without PCV2 vaccination; B: sows with mass vaccination; C: sow vaccination at 2–4 weeks pre-farrowing. Student’s *t*-test was used to assess differences in the presence of PCV2 MDA from suckling piglets born from different sow vaccination programs against PCV2. *p* values <0.05, <0.01, and <0.001 were considered statistically significant, highly significant, and very highly significant, respectively.

To examine the level of PCV2 MDA from different vaccination statuses of sows, the presence of PCV2 antibodies was calculated only from 3 to 4-week-old piglets without PCV2 vaccination. A total of 83 samples fit this criterion (Farms A, C, D, E, F, G, J, and K). As determined by commercial ELISA, the highest level of PCV2 MDA [ranging from 1.70 to 3.13, median 2.49, mean ± standard deviation (SD): 2.44 ± 0.34, coefficient of variation (CV): 13.77%] was observed in the sow vaccination against PCV2 at 2-4 weeks pre-farrowing group, followed by the mass vaccination group (ranging from 1.61 to 2.92, median 2.14, mean ± SD: 2.19 ± 0.35, CV 15.96%), and the difference was not statistically significant between those two groups (Figure 1). The level of PCV2 MDA in the sow without PCV2 vaccination group (ranging from 0.17 to 3.04, median 1.48, mean ± SD: 1.44 ± 0.70, CV 48.57%) was very highly significantly lower than that in the sow vaccination at 2-4 weeks pre-farrowing and mass vaccination groups (*p* < 0.0001). Additionally, the highest CV of PCV2 MDA was observed in the sow without PCV2 vaccination group.

### 3.2. Serodynamics of PCV2 Antibody in Pigs from Different Sow Vaccination Programs

**Figure 2:**
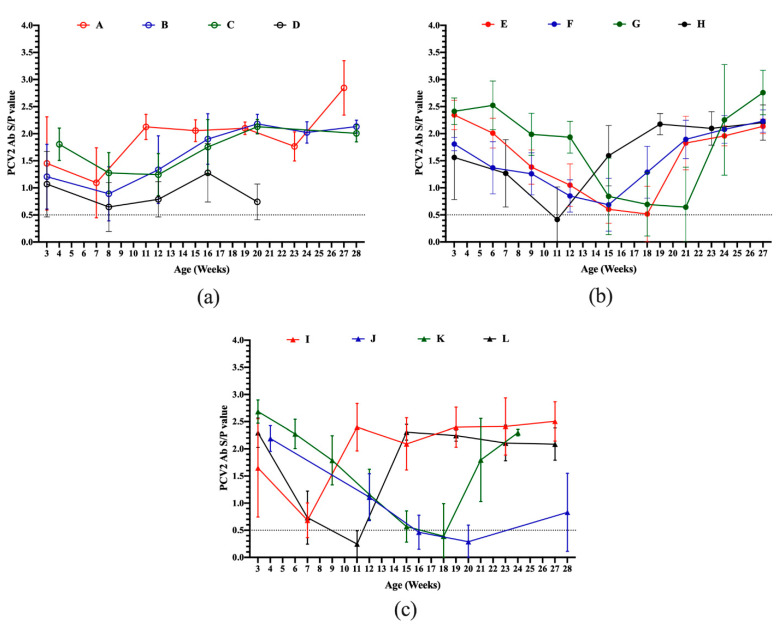
Serodynamic profile of porcine circovirus type 2 (PCV2) measured by BioCheck ELISA in different aged pigs born from different sow immunization programs against PCV2 from different farms. (**a**) sows without PCV2 vaccination (Farms A, B, C, and D); (**b**) sows with mass PCV2 vaccination (Farms E, F, G, and H); (**c**) PCV2 vaccination in sows at 2-4 weeks pre-farrowing (Farms I, J, K, and L).

The serodynamics of the PCV2 antibody in pigs during the suckling to slaughter stages from different sow vaccination programs were measured by ELISA and are shown in Figure 2. All pig herds, except Farm G, showed a decrease of PCV2 antibody levels during 3 to 4-week-old to 6 to 7-week-old of age, reaching the lowest levels of 0.65 to 1.24 S/P values at 7 to 12 weeks of age (Figure 2a), 0.41 to 0.69 S/P values at 11 to 21 weeks of age (Figure 2b) and 0.25 to 0.68 S/P values at 7 to 20 weeks of age (Figure 2c) in sows without PCV2 vaccination, sow vaccination at 2–4 weeks pre-farrowing and sow mass vaccination groups, respectively. After the lowest levels of PCV2 antibodies, the titers of PCV2 antibodies were dramatically increased (Figure 2a–c), providing evidence that pigs ever being exposed to PCV2 antigen. Only Farm G had slight seroconversion from 3 to 6 weeks old. Subsequently, antibody levels progressively declined, reaching the lowest levels of 0.64 S/P values at 21 weeks of age. These results indicated that pig herds showed slight or no seroconversion during the three to four weeks post-vaccination against PCV2.

### 3.3. Viral Dynamics of PCV2 Load in Pigs from Different Sow Vaccination Programs

**Figure 3:**
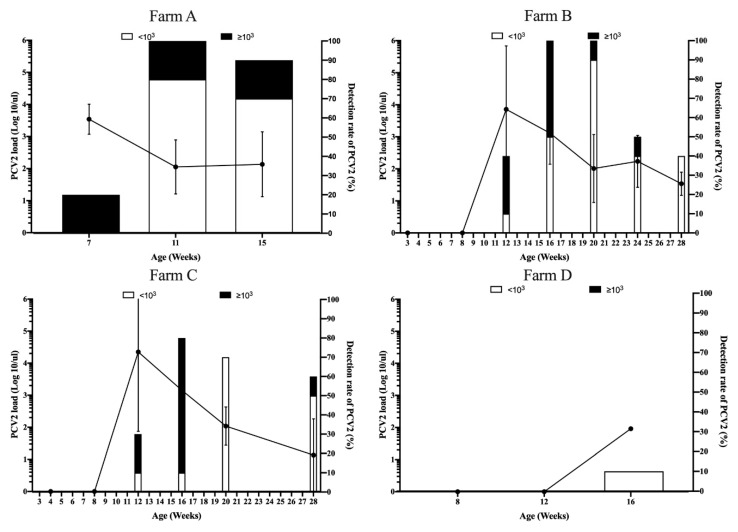
Viremia loads (line graph, left Y axis) and detection rate (bar chart, right Y axis) of porcine circovirus type 2 (PCV2) in pigs born from sows without PCV2 vaccination group, Farms A, B, C, and D. The error bars show the standard deviation (SD) of positive samples.

To elucidate the correlation between seroconversion and PCV2 natural infection, the PCV2 load during suckling to slaughter stages from all pig herds was quantitated by real-time PCR. The details of the detection rate of PCV2 and the range of PCV2 loads in different pig herds are shown in Table 2. High (≥10^3^ PCV2 genome copies/µl) or low PCV2 loads (<10^3^ PCV2 genome copies/µL) indicated PCVAD or subclinical PCV2 infection, respectively [5]. In the sow without vaccination against PCV2 group, high PCV2 viremia (≥10^3^ PCV2 genome copies/µL) was detected at 7 weeks old in 2 of 10 pigs in Farm A. Subsequently, ten of ten pigs (100%) and 9 of 10 pigs (90%) had PCV2 viremia at 11 and 15 weeks old, respectively (Figure 3 and Table 2). PCV2 viremia was first detected at 12 weeks old in 4 of 10 pigs (40%) and 3 of 10 pigs (30%) in Farms B and C, respectively (Table 2). High PCV2 loads were observed in 16 weeks old in both pig herds (Figure 3). Subsequently, PCV2 loads progressively declined until 28 weeks old (Figure 3). Interestingly, in Farm D, PCV2 viremia was only detected in one of 10 pigs (10%), with 10^1.96^ PCV2 genome copies/µl (Table 2).

**Figure 4:**
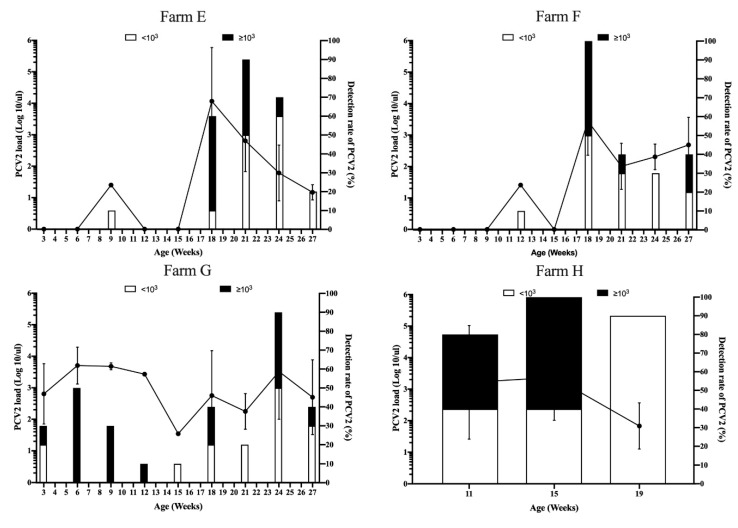
Viremia loads (line graph, left Y axis) and detection rate (bar chart, right Y axis) of porcine circovirus type 2 (PCV2) in pigs born from the sow mass PCV2 vaccination group, Farms E, F, G, and H. The error bars show the standard deviation (SD) of positive samples.

In the sow with mass vaccination against PCV2 group, PCV2 viremia was first detected at 9 and 12 weeks with 1 of 10 pigs (10%) in both Farm E and F, respectively (Table 2). High PCV2 loads were observed in 18 weeks old in both herds (Figure 4). Subsequently, PCV2 loads progressively declined until 27 weeks old (Figure 4). Surprisingly, PCV2 viremia was first detected at 3 weeks old in Farm G, which was the unvaccinated piglet stage (Figure 4 and Table 2). High PCV2 load was observed in 6-week-old pigs with 5 of 10 pigs (50%); then, the PCV2 load progressively declined until 15 weeks old (Figure 4 and Table 2). However, secondary and third PCV2 viremia were observed in 18 and 24 weeks old, respectively. In Farm H, high PCV2 viremia (≥ 10^3^ PCV2 genome copies/µl) was detected at 11 weeks old in 4 of 10 pigs (Table 2). Subsequently, ten of ten pigs (100%) and 9 of 10 pigs (90%) had PCV2 viremia at 15 and 19 weeks old, respectively (Figure 4).

**Figure 5:**
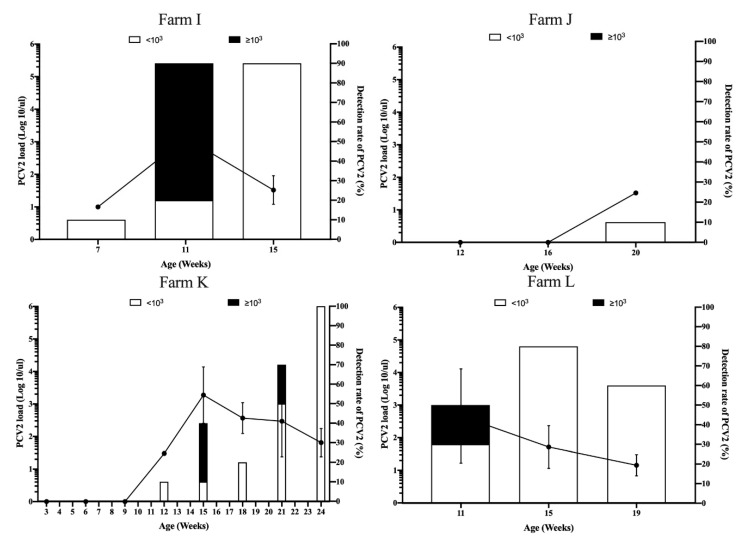
Viremia loads (line graph, left Y axis) and detection rate (bar chart, right Y axis) of porcine circovirus type 2 (PCV2) in pigs born from PCV2 vaccination in sows at 2–4 weeks pre-farrowing, Farms I, J, K, and L. The error bars show the standard deviation (SD) of positive samples.

In the sow vaccination against PCV2 in the pre-farrowing group, PCV2 viremia was first detected at 12 weeks with 1 of 10 pigs (10%) in Farm K (Figure 5 and Table 2). High PCV2 loads were observed in 15 and 21 weeks old (Figure 5 and Table 2). Ten of ten pigs (100%) had PCV2 viremia with subclinical infection of PCV2 in 24 weeks old (Figure 5 and Table 2). These results suggested that Farm K had high exposure to PCV2 at 15–24 weeks of age. However, PCV2 viremia was only detected in one of 10 pigs (10%), with 10^1.52^ PCV2 genome copies/µL in Farm J (Table 2). This may indicate that the mild exposure of PCV2 at 12–20 weeks of age in Farm J. Interestingly, in Farm I and L, high PCV2 viremia was only detected in 11 weeks old in both pig herds (Table 2). Subsequently, PCV2 loads were declined to low PCV2 loads (<10^3^ PCV2 genome copies/µL) (Figure 5).

Natural exposure to PCV2 should be considered starting at 11–12 weeks of age in the most analyzed pig farms. Regarding the production system (Table 1), natural exposure to PCV2 (as measured by real-time PCR) occurred in all herds, and seroconversion of PCV2 antibodies was subsequently observed in all herds in the present study (Figure 2). Taken together, the presence of PCV2 was observed during the window period for seroconversion in all herds.

## 4. Discussion

PCV2 is one of the major primary infectious agents causing mortality in PRDC in the field. PCVADs have caused enormous economic losses in industrial pork production worldwide [1]. Fortunately, effective vaccines have been developed within the past decade since the identification of PCV2, and these vaccines are currently used worldwide. However, a genotypic shift of PCV2 has been reported in several countries in recent years [18,20,21,22]. Additionally, despite widespread vaccination against PCV2, PCVADs or subclinical infection of PCV2 were also observed in the field in the United States [13] and European countries [23,24]. Thus, it is suggested that PCV2 still plays an important role in industrial pork production. This study explored the intersectional plane of correlation between antibodies and viremia in the natural exposure of PCV2 to shed light on the day-to-day situation in the field in Asia.

To compare the level of PCV2 MDA, it is not surprising that MDA was highly significantly lower in the sow without vaccination against PCV2 group than in the sow with mass or pre-farrowing vaccination groups. However, the highest CV of PCV2 MDA was also observed in the sow without PCV2 vaccination group. These results indicated that gilts and sows in pig herds have been naturally exposed to field viruses. Thus, sow vaccination against PCV2 should be a critical concern in the field. Our results indicated that a high level of PCV2 MDA was observed in the sow vaccination against PCV2 at 2–4 weeks in the pre-farrowing group, followed by the mass vaccination group. Fraile et al. [11] indicated a significant negative correlation between the level of PCV2 MDA and the level of PCV2 antibodies at 21 days post-vaccination. Therefore, what level of PCV2 MDA interferes with the efficacy of PCV2 vaccines in the clinic should be further studied. In addition to sow vaccination against PCV2 in the pre-farrowing and mass immunization, the efficacy of the post-farrowing vaccination (vaccination at weaning) is worthy of further investigation. To minimize the interference of the PCV2 MDA, the combination of vaccination in sows at mating and in piglets at 6–7 weeks old has been recommended [6,7]. However, porcine reproductive and respiratory syndrome virus (PRRSV) remains a major problem in the nursery stage (4–12 weeks of age) in most Asian counties [25,26,27], including Taiwan [28,29]. PRRSV infection can induce several immunosuppressive responses [30]. Therefore, the efficacy of delayed PCV2 immunization during the PRRSV susceptibility period should be further investigated.

To compare the sero- and viral dynamics of PCV2, despite the pig production system, sow and piglet vaccination programs, all pig herds, except the Farm G herd, showed a decrease of PCV2 total antibody levels during 3–4 to 6–7 weeks of age, reaching the lowest levels of 0.25 to 1.24 S/P values. The natural exposure of PCV2 as measured by real-time PCR occurred in all herds, and seroconversion of PCV2 antibodies was subsequently observed. Regarding natural exposure of PCV2 during the growing to fattening period, better protection against natural exposure of PCV2 under different types of vaccine, vaccination timing or two shots of PCV2 immunization worth to further investigated. This result is in accordance with a previous study; natural PCV2 infection can occur late in growing to fattening pigs, and viremia can last very long until slaughter [6]. One explanation for Farm G is that piglets that had already naturally been infected with field viruses before PCV2 vaccination (Figure 4 and Table 2). These results indicated that the BioChek ELISA profile of PCV2 can be used for the clinical evaluation of the severity of natural PCV2 exposure. Molecular techniques are not widely used due to high cost of infrastructure, equipment, maintenance, and professional labor in pig farm. In contrast, ready-to-use ELISA assay kits are commercially available for detecting and quantifying the specific antibodies conducted according to the manufacturer’s instructions. Previous studies reported the strongest correlation between the virus neutralization assay and ELISA assays [23,31]. Based on our results, pigs with an S/P ratio of 1 or lower are potentially susceptible to PCV2 infection. Taken together, use of the BioChek ELISA assay showed the level of antibodies and aided in the understanding and surveillance of the current PCV2 status in the field.

Consistent with previous field observations [6,11,12], no seroconversion was observed in any piglets at 3–4 weeks post-vaccination in the present study. Whether PRRSV infection in suckling piglets interferes with PCV2 seroconversion has been considered. Herein, we ruled out interference with PRRSV infection since PRRSV is classified as long-term stable and free in Farms K and D, respectively, monitored by the ADDC of NPUST. No seroconversion post-PCV2 immunization was observed in Farms K and D (Figure 2). Therefore, one explanation for the lack of seroconversion is the interference of PCV2 MDA post-PCV2 immunization. Similar findings have been reported and discussed under experimental or field conditions in the United States and European countries [4,6,7,11,12,13,14]. In addition to the humoral immune responses, all commercially available PCV2 vaccines can also induce cell-mediated immunity [4,5,6,7]. Thus, high PCV2 loads only occurred in some growing to fattening pigs in most pig herds, and very mild exposure of PCV2 in Farms D and J (Table 2) might imply that cell-mediated immunity alone will not fully eliminate PCV2 from the pig body. This may also be due to PCVADs having multifactorial syndromes and factors that are thought to affect the outcome of PCV2 natural infection [32]. Moreover, seroconversion post-immunization to determine whether a correlation exists between different types of PCV2 vaccines warrants further investigation.

## 5. Conclusions

Our study revealed the cross-sectional nature of the sero- and viral dynamics of PCV2 in twelve PCV2-contaminated pig herds in the field. The presence of PCV2 was observed during the window period for seroconversion in all herds. Natural exposure of PCV2 occurs in the growing to fattening period, and viremia can last until slaughter. Additionally, our findings indicate that using ELISA showed the level of antibodies and aided in the understanding and surveillance of the current PCV2 status in the field.

## Figures and Tables

**Table 1:** Details of the pig herd characteristics in this retrospective study.

Farm ID	Size ^a^	Production System	Country (Region) ^b^	PCV2 Vaccination Program (Vaccine)		Laboratory Examinations
				Sow	Piglet	
A	2000	One site	Pingtung (S)	Non-vaccination	3 weeks old (Ingelvac CircoFLEX^®^, Boehringer Ingelheim)	ELISA, qPCR
B	2800	Two-site	Tainan (S)	Non-vaccination	2 weeks old (Ingelvac CircoFLEX^®^, Boehringer Ingelheim)	ELISA, qPCR
C	1200	One site	Yunlin (C)	Non-vaccination	4 weeks old (Ingelvac CircoFLEX^®^, Boehringer Ingelheim)	ELISA, qPCR
D	350	One site	Tainan (S)	Non-vaccination	3 weeks old (Ingelvac CircoFLEX^®^, Boehringer Ingelheim)	ELISA, qPCR
E	400	One site	Yunlin (C)	Mass vaccination, 2 times/year (Ingelvac CircoFLEX^®^, Boehringer Ingelheim)	3 weeks old (Ingelvac CircoFLEX^®^, Boehringer Ingelheim)	ELISA, qPCR
F	2000	Three-site	Yunlin (C)	Mass vaccination, 2 times/year (Ingelvac CircoFLEX^®^, Boehringer Ingelheim)	3 weeks old (Ingelvac CircoFLEX^®^, Boehringer Ingelheim)	ELISA, qPCR
G	550	One site	Yunlin (C)	Mass vaccination, 2 times/year (Ingelvac CircoFLEX^®^, Boehringer Ingelheim)	3 weeks old (Ingelvac CircoFLEX^®^, Boehringer Ingelheim)	ELISA, qPCR
H	2000	One site	Pingtung (S)	Mass vaccination, 2 times/year (Ingelvac CircoFLEX^®^, Boehringer Ingelheim)	2 weeks old (Ingelvac CircoFLEX^®^, Boehringer Ingelheim)	ELISA, qPCR
I	1900	One site	Pingtung (S)	3 weeks pre-farrowing (Ingelvac CircoFLEX^®^, Boehringer Ingelheim)	2 weeks old (Ingelvac CircoFLEX^®^, Boehringer Ingelheim)	ELISA, qPCR
J	3300	Two-site	Yunlin (C)	4 weeks pre-farrowing (Ingelvac CircoFLEX^®^, Boehringer Ingelheim)	4 weeks old (Ingelvac CircoFLEX^®^, Boehringer Ingelheim)	ELISA, qPCR
K	350	One site	Miaoli (C)	4 weeks pre-farrowing (Circovac^®^, Merial)	3 weeks old (Ingelvac CircoFLEX^®^, Boehringer Ingelheim)	ELISA, qPCR
L	2000	One site	Pingtung (S)	2 weeks pre-farrowing (Ingelvac CircoFLEX^®^, Boehringer Ingelheim)	2 weeks old (Ingelvac CircoFLEX^®^, Boehringer Ingelheim)	ELISA, qPCR

^a^ Number of sows. ^b^ Region of Taiwan. S: Southern Taiwan; C: Central Taiwan.

**Table 2:** Percentage of porcine circovirus type 2 (PCV2) detection and PCV2 loads in different aged pigs.

Farm	Age (Weeks)																
	3	4	6	7	8	9	11	12	15	16	18	19	20	21	24	27	28
A				20 (2/10); 3.21–3.87			100 (10/10); 1.11–3.29		90 (9/10); 1.00–3.60								
B	0 (0/10) ^a^				0 (0/10)			40 (4/10); 1.38–6.11		100 (10/10); 2.11–4.89			100 (10/10); 1.04–4.28		50 (5/10); 1.32–3.38		40 (4/10); 1.08–1.86
C	^b^	0 (0/10)			0 (0/10)			30 (3/10); 1.76–6.70		80 (8/10); 1.28–3.91			70 (7/10); 1.36–2.96				60 (6/10); 1.18–3.32
D					0 (0/10)			0 (0/10)		10 (1/10) 1.96							
E	0 (0/10)		0 (0/10)			10 (1/10); 1.40		0 (0/10)	0 (0/10)		60 (6/10); 1.57–6.56			90 (9/10); 1.70–4.55	70 (7/10); 1.00–3.63	20 (2/10); 1.00–1.34	
F	0 (0/10)		0 (0/10)			0 (0/10)		10 (1/10); 1.41	0 (0/10)		100 (10/10); 2.17–4.92			40 (4/10); 1.41–3.01	30 (3/10); 1.90–2.71	40 (4/10); 1.52–3.50	
G	30 (3/10); 1.79–3.69		50 (5/10); 3.11–4.56			30 (3/10); 3.56–3.77		10 (1/10); 3.43	10 (1/10); 1.54		40 (4/10); 1.20–3.97			20 (2/10); 1.85–2.65	90 (9/10); 1.96–5.50	40 (4/10); 1.30–4.18	
H							80 (8/10); 1.13–6.11		100 (10/10); 1.54–5.60			90 (9/10); 1.00–2.93					
I				10 (1/10); 1.00			90 (9/10); 1.43–3.73		90 (9/10); 1.00–2.19								
J								0 (0/10)		0 (0/10)			10 (1/10); 1.52				
K	0 (0/10)		0 (0/10)			0 (0/10)		10 (1/10); 1.48	40 (4/10); 2.08–4.11		20 (2/10); 2.23–2.90			70 (7/10); 1.11–4.08	100 (10/10); 1.15–2.46		
L							50 (5/10); 1.00–4.69		80 (8/10); 1.00–2.62			60 (6/10); 1.00–1.81					

^a^ % of PCV2-positive (positive/all tested); PCV2 loads (minimum-maximum); ^b^ The empty cells corresponding to samples that were not collected or those that were not detected using qPCR.

## Supplementary Materials

The following are available online at https://www.mdpi.com/2076-393X/8/2/339/s1, Table S1: Details of case numbers of each pig herd at different ages.
