# Supplementary material for: Cross-Sectional Study on the Sero- and Viral Dynamics of Porcine Circovirus Type 2 in the Field
Source: Vaccines (Basel). 2020 Jun 26;8(2):339. doi: 10.3390/vaccines8020339 (PMC7350207; doi:10.3390/vaccines8020339)
Supplement: Supplementary file 1 [file vaccines-08-00339-s001.pdf]

Table S1. Details of case numbers of each pig herd at different ages.

Farm	Age (weeks)																		Total
	3	4	6	7	8	9	11	12	15	16	18	19	20	21	23	24	27	28	
A	13			10			10		10			10			10		10		73
B	10				10			10		10			10			10		10	70
C		10			10			10		10			10					10	60
D	10				10			10		10			10						50
E	10		10			10		10	10		10			10		10	10		90
F	10		10			10		10	10		10			10		10	10		90
G	10		10			10		10	10		10			10		10	10		90
H	13			10			10		10			10			10		9		72
I	15			10			10		10			10			10		10		75
J		10						10		10			10					10	50
K	10		10			10		10	10		10			10		10			80
L	15			10			10		10			10			10		10		75
Total	116	20	40	40	30	40	40	80	80	40	40	40	40	40	40	50	69	30	875
